# GQ1b-Positive Pharyngeal-Cervical-Brachial Variant of Guillain-Barré Syndrome Presenting With Pseudoathetosis and Pseudodystonia in a 47-Year-Old Filipino Female: A Case Report

**DOI:** 10.7759/cureus.80697

**Published:** 2025-03-17

**Authors:** Cybele Liana D Go, Jon Stewart H Dy, Raymond L Rosales

**Affiliations:** 1 Neurology, Institute for Neurosciences, St. Luke's Medical Center, Global City, PHL; 2 Neurology, Institute for Neurosciences, St. Luke's Medical Center, Quezon City, PHL; 3 Neurology, St. Luke's Medical Center, Quezon City, PHL; 4 Neurology, Movement Disorders, and Neurophysiology, University of Santo Tomas Hospital, Manila, PHL; 5 Neurology, Movement Disorders, and Neurophysiology, Institute for Neurosciences, St. Luke's Medical Center, Quezon City, PHL

**Keywords:** gq1b ganglioside antibody, guillain-barré syndrome, intravenous immunoglobulin, pharyngeal-cervical-brachial variant, pseudoathetosis, pseudodystonia

## Abstract

Guillain-Barré syndrome (GBS) is the most common cause of acute ascending symmetrical paralysis in clinical practice. One of its regional variants is the pharyngeal-cervical-brachial (PCB) variant associated with antibodies against GT1a and GQ1b ganglioside. Pseudoathetosis and pseudodystonia have not yet been reported in this variant. In this study, we report a case of a 47-year-old Filipino female who developed rapidly progressive dysarthria, bibrachial paralysis, and ascending dysesthesias, with pseudoathetosis, or abnormal writhing movements of the extremities, and pseudodystonia, or abnormal postures. Cerebrospinal fluid analysis demonstrated albuminocytologic dissociation, and electrodiagnostic (EDX) studies showed evidence of proximal nerve involvement with predominant late response abnormalities consistent with a demyelinating neuropathy. Ganglioside antibody testing revealed seropositivity for anti-GQ1b. She was given a five-day course of intravenous immunoglobulin (total of 2 grams per kilogram) and underwent physical, speech, and occupational therapy. She was discharged with residual neurologic deficits rendering her bed-bound and assisted for activities of daily living. Two months after treatment, clinical outcome in our patient after intravenous immunoglobulin and regular physical rehabilitation showed complete recovery without any neurologic sequelae.

## Introduction

Guillain-Barré syndrome (GBS) is an acute immune-mediated neurologic disease with systemic and regional variants. It is caused by the presence of antibodies that attack the different components of axons and their surrounding myelin sheath [1]. The pharyngeal-cervical-brachial (PCB) variant is a regional variant of GBS characterized by acute onset weakness of the oropharyngeal, neck, and shoulder muscles with swallowing dysfunction and arm hyporeflexia in the absence of ataxia, disturbed consciousness, and prominent leg weakness [2]. Pseudoathetosis (abnormal writhing movements, usually of the fingers) and pseudodystonia (abnormal postures and repetitive movements) have yet to be described as consistent features in this variant. The diagnosis of the PCB variant of GBS is primarily based on clinical presentation and electrophysiologic studies [2]. Supportive criteria for its diagnosis include the presence of GT1a or GQ1b ganglioside antibodies [2,3]. Despite the availability of ganglioside antibody testing, there is no local data regarding the incidence of the PCB variant of GBS. In this study, we report a patient who presented with acute onset dysarthria, bibrachial paralysis, dysesthesias, and, notably, prominent features of pseudoathetosis and pseudodystonia. The patient made a full recovery without any residual neurological deficits. She is the first locally documented case of a seropositive anti-GQ1b PCB variant of GBS.

## Case presentation

The patient was a 47-year-old female from the Philippines with a prior history of controlled stage I essential hypertension maintained on telmisartan. She worked as a financial adviser and was independent in all activities of daily living. She had three prior COVID-19 vaccination doses and no previous COVID-19 infection. She had no history of fever, cough, nasal congestion, or diarrhea prior to the onset of symptoms.

She presented with acute-onset generalized body weakness and paroxysmal episodes of positional vertigo. Despite her symptoms, she was still ambulatory and able to perform daily activities. On the second day of illness, she developed dysesthesias over both feet. She sought consultation at a local hospital, where she had an unremarkable physical examination, laboratory workup, and plain cranial computed tomography (CT) scan results. She was managed as a case of transient ischemic attack and started on antiplatelet therapy. Despite the medication, there was no improvement in symptoms. On the third day of illness, dysesthesias worsened and ascended to the bifacial area. She now complained of bilateral ptosis, facial diplegia, dysarthria, and bibrachial paresis and required assistance during ambulation. She then consulted at our institution for further management.

Upon assessment at our institution, she had stable vital signs and an unremarkable systemic physical examination. Neurologic examination revealed severe bilateral ptosis, external ophthalmoparesis, and facial diplegia, a symmetrical but weak palatal elevation and gag reflex, and moderate dysarthria and hypernasal speech. She was fully awake and oriented to place, person, and time. Cortical examination, visual acuity, and pupillary reflexes were intact. Examination of the extremities showed generalized flaccidity and bibrachial paresis (motor strength of both upper extremities at 3/5 and both lower extremities at 4+/5). There was a marked pseudoathetosis of the bilateral upper extremities and pseudodystonia of the bilateral lower extremities. Sensory examination showed decreased vibration and proprioception, dysesthesias, and a sensory ataxia over both upper and lower extremities. Light touch, pain, and temperature sense were intact. There was no nystagmus, titubation, or saccadic dysmetria. There was generalized areflexia and no nuchal rigidity.

Laboratory examinations (Table 1) showed mild anemia and mild leukocytosis with neutrophilic predominance. Non-contrast cranial magnetic resonance imaging (MRI) results were unremarkable for acute strokes or mass lesions. There were no lesions seen in the thalamus, basal ganglia, or brainstem. Electrodiagnostic (EDX) studies (Tables 2-4) done on the fourth day of illness showed bilaterally reduced direct facial compound muscle action potential amplitudes with associated wave dispersion bilaterally, absent early and late components of the blink reflex, absent late responses (tibial H reflexes), and bilateral tibial F wave responses with more than expected wave dispersion (Table 4). Electromyography showed reduced recruitment but otherwise had no denervation potentials and normal waveform morphology (Table 5). These findings were consistent with craniopathies involving both facial nerves and proximal neuropathies involving both lower limbs.

**Table 1:** Serum tests. pCO2: partial pressure of carbon dioxide; pO2: partial pressure of oxygen; HCO3: bicarbonate.

Test	Results	Reference value
Hemoglobin	11.3 g/dL	13.0-17.0 g/dL
Hematocrit	35.4%	40.0-52.0%
White blood cell count	12,290 mm^3^	4,800-10,800 mm^3^
Neutrophils	85%	40-74%
Lymphocytes	10%	19-48%
Platelet count	329,000/mm^3^	150,000-400,000/mm^3^
Creatinine	0.70 mg/dL	0.7-1.3 mg/dL
Sodium	140 mmol/L	136-145 mmol/L
Potassium	3.6 mmol/L	3.5-5.1 mmol/L
Alanine transferase	27 U/L	10-49 U/L
Aspartate transferase	25 U/L	0-34 U/L
Arterial blood gas pH	7.44	7.35-7.45
Arterial blood gas pCO2	36 mmHg	35-45 mmHg
Arterial blood gas pO2	88 mmHg	80-100 mmHg
Arterial blood gas HCO3	25.3 mmol/L	22-26 mmol/L

**Table 2:** Facial nerve stimulation study.

Muscle	Parameter	Left	Right
Orbicularis oculi	Distal latency	3.3 ms	3.3 ms
	Amplitude	3.5 mV	3.8 mV
Nasalis	Distal latency	3.1 ms	3 ms
	Amplitude	2.6 mV	2.1 mV
Frontalis	Distal latency	3.1 ms	3 ms
	Amplitude	0.7 mV	0.7 mV
Orbicularis oris	Distal latency & amplitude	No response	No response

**Table 3:** Blink reflex studies.

Supraorbital nerve stimulation	Ipsilateral responses latency		Contralateral response latency
	R1	R2	R2
Left	No response	No response	No response
Right	14.5 ms	39.6 ms	41.4 ms

**Table 4:** Motor nerve conduction studies of the lower extremities.

Nerve	Site	Amplitude (mV)	Conduction velocity (m/s)	Distal latency (ms)	F wave (ms)	H reflex (ms)
Left common peroneal	Ankle	5.1	42 (leg), 55 (across knee)	2.4	Not done	Not done
	Knee	5				
	Above knee	4.9				
Right common peroneal	Ankle	11.4	49 (leg), 48 (across knee)	3.2	Not done	Not done
	Knee	10.8				
	Above knee	10.8				
Left posterior tibial	Ankle	19.2	46	4.3	42.6	No response
	Knee	19				
Right posterior tibial	Ankle	19	48	4.8	47.6	No response
	Knee	14.9				

**Table 5:** Electromyography of the facial area and upper and lower extremities.

Muscles		Insertional activity	Spontaneous activity			Motor unit potential
			Fibrillation	Fasciculation	Positive sharp waves	
Facial muscles	Left orbicularis oculi	Normal	-	-	-	Reduced recruitment
Upper extremity muscles	Left first dorsal interossei	Normal	-	-	-	Normal
Lower extremity muscles	Left tibialis anterior	Normal	-	-	-	Reduced recruitment
	Left gastrocnemius (medial)	Normal	-	-	-	Normal

Given these findings, she was managed as a case of acute distal symmetric polyneuropathy secondary to an anti-GQ1b-positive PCB variant of GBS. She had a Modified Erasmus GBS Outcome Score (mEGOS) of 5 and an Erasmus GBS Respiratory Insufficiency Score (EGRIS) of 5. She was admitted to the neurocritical care unit and started on a single course of intravenous immunoglobulin (2 grams per kilogram over the course of five days) and physical rehabilitation. A cerebrospinal fluid analysis (Table 6) done on the third day of hospitalization and on the sixth day of illness showed albuminocytologic dissociation. Serum ganglioside antibody testing (Table 7) showed seropositivity for anti-GQ1b.

**Table 6:** Cerebrospinal fluid analysis results.

Test	Results	Reference value
Opening pressure	10 cm H2O	8-18 cm H2O
Closing pressure	8 cm H2O	8-18 cm H2O
Gross description	Clear	None
Total cell count	0 cells/mm^3^	0-5 cells/mm^3^
CSF-serum glucose ratio	0.62	0.5-0.6
Protein	71 mg/dL	8-32 mg/dL
Culture and sensitivity, acid-fast bacilli, Cryptococcal Antigen Latex Agglutination System, film array	Negative	Negative
Cell block and cytology	Negative	Negative

**Table 7:** Antiganglioside antibody testing results. Results for ganglioside antibody testing showed seropositivity for anti-GQ1b IgM and IgG.

Ganglioside antibodies, IgG-IgM	Results (IV)	Reference value (IV)
Asialo-GM1 antibodies, IgG-IgM	23	0-50
GM1 antibodies, IgG-IgM	16	0-50
GM2 antibodies, IgG-IgM	26	0-50
GD1a antibodies, IgG-IgM	Not done due to unsatisfactory reagent performance	0-50
GD1b antibodies, IgG-IgM	18	0-50
GQ1b antibodies, IgG-IgM	89	0-50

After intravenous immunoglobulin administration, her neurologic status gradually improved. There was lesser bilateral ptosis, ophthalmoparesis, facial diparesis, and moderate dysarthria. There was persistence of pseudoathetosis, bibrachial paresis, and dysesthesias. Neither dysautonomia nor respiratory difficulties were observed. Follow-up neurophysiological studies done on the 22nd day of illness showed persistent absent bilateral late responses (tibial H reflexes) but otherwise normal nerve conduction studies of the upper and lower extremities. Significant improvement was seen in the follow-up blink reflex and facial nerve simulation studies that now showed normal distal latencies and compound muscle action potential amplitudes of the facial muscles tested, and normal early and late components of supraorbital nerve stimulation.

On discharge, she had mild bilateral ptosis and ophthalmoparesis, facial diparesis, moderate dysarthria, improved motor strength of the bilateral upper extremities at 3+/5 and bilateral lower extremities at 4+/5, lesser pseudoathetosis, and generalized hyporeflexia. Physical rehabilitation was continued after discharge. Two months after treatment, she showed complete resolution of all her neurologic deficits except for the generalized hyporeflexia, and she was able to resume all her activities of daily living.

## Discussion

The PCB variant of GBS is the second most frequent regional variant of the disease. It has an incidence of 0.07-0.25 per 100,000 population and a slight male predominance (1.3:1), with a median age of onset at 43 years old. Antecedent respiratory tract and gastrointestinal tract infections have been observed prior to the onset of symptoms [2,3]. Our patient’s demographic and clinical profile is consistent with the available literature, with a few exceptions. First, there was no identified antecedent illness, which is seen in one-third of the cases of GBS reported in the literature [1]. Second, there was the presence of ascending dysesthesia, which is an uncommon initial symptom in this variant [2,3]. Third, the patient presented with pseudoathetosis and pseudodystonia. Pseudoathetosis is a movement disorder presenting with continuous, involuntary slow writhing movements of the distal extremities. It is usually caused by impaired proprioception. Pseudodystonia is characterized by abnormal postures and repetitive movements mimicking dystonia. It is associated with a wide range of both non-neurological disorders of the musculoskeletal system and disorders of sensory or motor pathways. Therefore, the presence of associated neurological findings (such as proprioceptive sense in the present case) in the affected body part is key toward reaching a diagnosis of pseudoathetosis and pseudodystonia [4]. Though its incidence rate in GBS has not yet been studied, pseudoathetosis has only been reported in cases of pure sensory GBS and sensory ataxic GBS with IgG anti-GM1 antibodies [5,6].

Cerebrospinal fluid (CSF) albuminocytologic dissociation and neurophysiologic evidence of neuropathy in the neck and upper extremities are supportive features of the disease [7]. In a case series study of PCB variants of GBS, CSF results were abnormal in only 40% of patients, most commonly showing only slightly elevated protein [3]. In our patient, albuminocytologic dissociation was seen within the first week of the disease.

Neurophysiologic studies may be normal when performed within one week of illness onset. The addition of facial nerve and blink reflex studies is, therefore, useful in the early diagnosis of GBS and in clinically symptomatic patients with ophthalmoplegia and bulbar dysfunction [1,2]. EDX studies performed on our patient showed the absence of neuropathy in the upper extremities but the presence of neuropathy in the lower extremities, whereas currently reported cases of this variant present with upper extremity neuropathy [2].

Seropositivity for anti-GQ1b and anti-GD1a underlies the molecular mimicry mechanism associated with the disease [7,8]. Both antibodies are found in the neck, arm, and leg, while the anti-GQ1b antibody is also found in the oculomotor nerves, glossopharyngeal and vagal nerves, reticular formation, and cerebellum. The known clinical spectrum of an “anti-GQ1B syndrome” includes Miller Fisher syndrome, Bickerstaff brainstem encephalitis, acute ophthalmoplegia without ataxia, acute vestibular syndrome, optic neuropathy with disc swelling, and acute sensory ataxic neuropathy. The Miller Fisher variant presents with a triad of ophthalmoplegia, ataxia, and areflexia, and is a common differential for the PCB variant [9]. However, our patient presented with prominent bibrachial paralysis, which is more consistent with the PCB variant of GBS. Notably, pseudoathetosis and pseudodystonia have yet to be described as consistent features in either variant [9,10].

The administration of intravenous immunoglobulin or plasma exchange within four weeks from illness remains the standard of treatment in patients with GBS, regardless of the variant, with both treatment options having equal efficacy [7,11]. Our patient was given a single course of intravenous immunoglobulin for a total of 2 grams given over five days.

The mEGOS and EGRIS predict outcomes in GBS [12]. The pure PCB variant of GBS was more likely to require intubation, especially if there was severe bulbar involvement [3]. Based on our patient’s mEGOS and EGRIS, she had a 10-15% probability of walking independently at six months [12] and a 20-30% probability of requiring ventilatory support within one week of hospitalization, respectively [13]. Despite the severity of her symptoms and bulbar dysfunction, our patient did not require ventilatory support. The findings in our patient corroborate existing literature that the seropositive PCB variant of GBS had a better clinical outcome compared to seronegative patients [2,3,8].

A limitation of our study was that no follow-up ganglioside antibody titer was obtained. Antibody persistence as a possible treatment biomarker has not been studied in this variant but has been used in patients with other variants of GBS [14]. Although the current use of antibody testing is for the diagnosis of the PCB variant of GBS, documenting seroconversion could prove invaluable in outcome prediction and risk of relapse [11,14]. At the time of writing, there are no existing clinical practice guidelines for the use of ganglioside antibodies as a treatment biomarker in the disease.

## Conclusions

In summary, we presented the case of a 47-year-old Filipino female who presented with an acute, predominantly bulbar, ocular, and bibrachial weakness on top of a distal symmetric polyneuropathy secondary to an anti-GQ1b-positive PCB variant of GBS. She is the first local documentation of this variant and presented with prominent pseudoathetosis and pseudodystonia. This case highlights the importance of a high clinical suspicion and thorough neurological examination in patients presenting with abnormal movements. This information is invaluable in facilitating the early diagnosis of critical diseases such as GBS. Additionally, while the benefit of antibody testing has been extensively studied in the systemic variants of GBS, its role in the prognostication of the regional GBS variants is still unclear. Further studies are needed to improve the existing clinical guidelines to aid in early diagnosis, treatment, and clinical outcome prediction, thereby reducing morbidity and mortality associated with the disease.
